# Inequality, well-being, and the problem of the unknown reporting function

**DOI:** 10.1073/pnas.2217750119

**Published:** 2022-12-06

**Authors:** Caspar Kaiser, Andrew J. Oswald

**Affiliations:** ^a^University of Oxford, Oxford OX1 3TD, United Kingdom; ^b^University of Warwick, Coventry CV4 7AL, United Kingdom

Every politician, in every nation and in every era of history, eventually has to face a complex and emotive question. Should I try to redistribute money from my richer citizens to my poorer citizens? If so, by how much? This is a timeless issue. The appropriate answer to the question turns crucially on a claim that goes back hundreds of years to, for example, the philosopher Jeremy Bentham: “All inequality is a source of evil—the inferior loses more in the account of happiness than by the superior is gained.” (1) In an ideal world, a hypothesis of this sort would be tested in a giant randomized controlled trial (RCT), perhaps funded by a body such as the National Science Foundation of the United States. However, no funding body is likely to provide the necessary millions of dollars to run that experiment-until now. In a remarkable and important contribution to conceptual science and practical public policy, Ryan Dwyer and Elizabeth Dunn (2) have—with the help of millionaire donors—run an RCT that comes close to that ideal.

Before the paper by Dwyer and Dunn, a long literature, going back to Richard Easterlin and Edward Diener among others, had established that there is an upward-sloping, although curved, relationship between being richer and saying in a survey that you feel happier. Fig. 1 gives a modern version of the famous pattern.

**Fig. 1.**
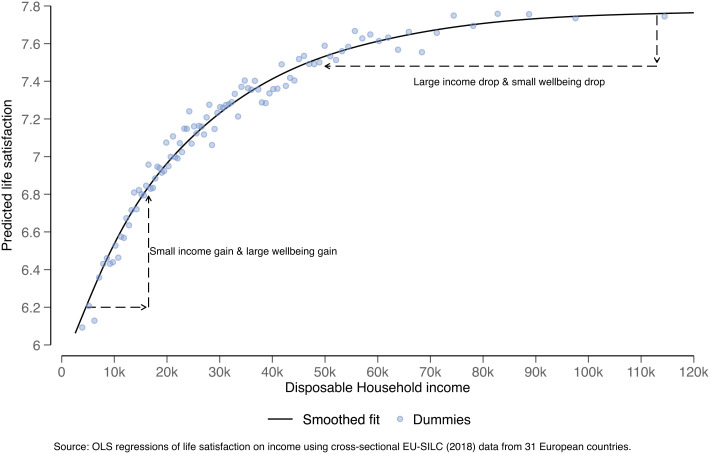
Illustration of the potential well-being gains from redistribution. This figure illustrates why redistribution can in principle raise well-being when the income-to-well-being relationship is curved in a concave way. The bottom left demonstrates that a relatively small income increase for low-income individuals can result in a large well-being gain. Contrastingly, as shown in the top right, a relatively large drop in incomes for high-income individuals will result in a relatively small well-being drop. The black line plots the result of an OLS regression of life satisfaction (measured on a 0–10 scale) on disposable household incomes. Incomes are here entered as a flexible spline. The dots show the result of a separate regression in which dummies are fitted for each percentile of the European income distribution. Both these regression equations control for age and age squared, gender, household size, number of children, education, employment status, migrant status, as well as country dummies. The data are cross-sectional, are representative of 31 European countries, and are sourced from the 2018 wave of the European Union Statistics on Income and Living Conditions survey (EU-SILC). Income is measured in Euros and after tax.

## The Breakthrough Contribution of Dwyer and Dunn Is to Place This Association on Firm Causal Foundations

Dwyer and Dunn create an experiment in which assignment to treatment is random. The authors’ work is an example of a more general movement in modern social science in which earlier correlational research is checked with experimental and quasiexperimental designs (e.g., ref. 3). They add also to an emerging causal literature on cash transfers and well-being in low- and middle-income countries (summarized in, e.g., ref. 4). Dwyer and Dunn deliberately field the same kind of cash transfer in several different economic contexts. In this way, they connect two kinds of literature and demonstrate that cash transfers do indeed improve recipients’ self-reported well-being across a wide variety of settings. There are large causal effects that persist over at least a 6-mo period.

Crucially, in the authors’ experimental research, it is the poorer recipients of (randomly assigned) money who show larger psychological gains. From that, the authors conclude that a reduction in income inequality would raise overall well-being. Their argument has two parts. First, drawing on prior research in the literature (5), Dwyer and Dunn note that the well-being loss incurred by the (millionaire) donors of the cash is likely to be much smaller than the total well-being gains among recipients. Second, the authors discover that the cash transfers’ estimated effect-size appears to decline in a very particular way. It does so linearly in the log of recipients’ income. This is evidence of a curved (concave) well-being-to-income relationship. In other words, as Bentham hypothesized, there are diminishing marginal returns to income.

As is well known—and shown in Fig. 1—a concave relationship between income and well-being entails that wider income inequality will pull down the average level of well-being. This is a corollary of a theorem known as Jensen’s Inequality in mathematics.

## Three Scientific Complications Now Stand Out

One complication—formally recognized more than half a century ago (6), but it continues to be hotly debated in political life—is that tax-funded redistribution may distort incentives and thereby dampen economic growth. If so, redistribution could act to raise well-being via a more equal income distribution but at the same time could lower well-being by decreasing the size of the ‘pie’. Assessing the relative importance of these countervailing forces—as discussed in conventional economics courses—remains a priority.

Loss aversion is a second complication. Income losses are known to loom larger than gains. In the short term, therefore, the well-being losses of those who are taxed may exceed the gains of net recipients. Based on earlier work (7), Dwyer and Dunn suggest that voluntarily giving money away can increase people’s well-being. Whether that is also true in the case of forced redistribution is an open question. It may eventually be possible to answer that by building on current studies of loss aversion (such as refs. 8 and 9).

## The Third Complication is the Most General

It clearly seems as though Dwyer and Dunn give causal evidence for a curved well-being shape. Yet, as they mention in passing, although they do not elaborate, their paper depends on an untested assumption. It is that the relationship between reported well-being and the underlying actual well-being is linear. Whilst scattered work hints at possible linearity (10–13), nobody currently knows whether that is true. It depends on how humans use language when they answer happiness kinds of questions. This is a particular version of a generalized difficulty outlined in a recent piece by Bond and Lang (14) and previously discussed in Oswald (12). The earlier literature on psychophysics also grappled with this (15). Without knowledge of the ‘reporting function’, we cannot be sure that we can treat well-being data as ratio-scale measurements, which is required for statements like “three times more happiness”.

“Dwyer and Dunn deliberately field the same kind of cash transfer in several different economic contexts. In this way, they connect two literatures and demonstrate that cash transfers do indeed improve recipients’ self-reported wellbeing across a wide variety of settings.”

Let us make the (presumably reasonable) assumption that happier people tend to also report themselves in surveys as happier. Although this is sometimes debated too (14, 16–18), we can then hope to learn about the direction of different influences, like bad illness and higher income, upon subjective well-being. However, it takes more demanding assumptions about the nature of people‘s answers if we are to make robust inferences about curvature, and, more generally, about the relative magnitudes of different influences on well-being. For example, imagine that the income-to-well-being relationship is actually linear and not curved. Yet, now, suppose that people, as they feel cheerier, mark themselves as happier on a questionnaire scale in a way in which they are intrinsically reluctant to approach the upper possible levels on the questionnaire form (the 9 and 10 on a 1–10 scale, say), then the reporting function itself is curved.^*^ Such nonlinearity might occur if respondents treat Likert scales as akin to academic grading systems (19), in which the very top scores are, as in many countries, almost impossible to attain. Another source of nonlinearity might be that people’s potential for suffering may be greater than their capacity for happiness. If so, the bottom scores would cover a wider range of feelings than the top scores. The substantive conclusion in Dwyer and Dunn could be affected in either case.

In a diagram like Fig. 1, the empirical shape linking income and reported well-being actually bundles up two relationships (two equations) into one (12). One relationship is how money affects underlying well-being; the second relationship is how humans report their feelings given any particular level of well-being. There are two relationships, not one—both of these could be curved; neither could be curved; or only one could be curved. Since the welfare-increasing effects of redistribution rely on the idea of that curvature being genuinely about people’s happiness from money, rather than about linguistic cautiousness in people’s use of numbers, we cannot know for certain the degree to which income redistribution might raise actual well-being.

We would like to emphasize that our instinct is that the authors’ finding is correct and that Dwyer and Dunn have written a superb paper of lasting importance. Further research in this area will still be appropriate. It may include qualitative work, perhaps with in-depth interviews on respondents’ scale-use, as well as quantitative work that would systematically map subjective responses to observable cardinal quantities (as in refs. 12 and 15). Currently, the reporting-function problem is fundamental, little recognized, and so far unsolved.

## Footnotes

^*^The issue of curved reporting functions is related to, but is conceptually distinct from, concerns about ceiling and floor effects. In the latter case, there is an excess—rather that sparsity—in the share of respondents who select the top and bottom categories. This is often interpreted as a form of data censoring (19).
